# Supplementary material for: Cancer inpatients with COVID-19: A report from the Brazilian National Cancer Institute
Source: PLoS One. 2020 Oct 26;15(10):e0241261. doi: 10.1371/journal.pone.0241261 (PMC7588058; doi:10.1371/journal.pone.0241261)
Supplement: S1 Table — (DOCX) [file pone.0241261.s001.docx]

S1 Table. Data on cancer by site

| **Cancer type** | **Frequency** | **Percent** |
| --- | --- | --- |
| Breast | 40 | 22,1 |
| Lymphoma | 20 | 11 |
| Colorectal | 14 | 7,7 |
| Cervical cancer | 12 | 6,6 |
| Central nervous system | 11 | 6,1 |
| Leukemia | 10 | 5,5 |
| Prostate | 10 | 5,5 |
| Lung | 7 | 3,9 |
| Stomach | 6 | 3,3 |
| Pharynx | 5 | 2,8 |
| Endometrium | 5 | 2,8 |
| Larynx | 4 | 2,2 |
| Multiple myeloma | 4 | 2,2 |
| Urothelium | 3 | 1,7 |
| Ovary | 3 | 1,7 |
| Skin | 3 | 1,7 |
| Kidney | 3 | 1,7 |
| Ependymoma | 2 | 1,1 |
| Pancreas | 2 | 1,1 |
| Unknown primary site | 2 | 1,1 |
| Retinoblastoma | 2 | 1,1 |
| Sarcoma | 2 | 1,1 |
| Vulva | 2 | 1,1 |
| Aplastic anemia | 1 | 0,6 |
| Hepatocellular carcinoma | 1 | 0,6 |
| Dermatofibrosarcoma | 1 | 0,6 |
| Tongue | 1 | 0,6 |
| Melanoma | 1 | 0,6 |
| Penis | 1 | 0,6 |
| Retroperitoneum | 1 | 0,6 |
| Thyreoid | 1 | 0,6 |
| Gall bladder | 1 | 0,6 |
| Total | 181 | 100 |
