# Supplementary material for: Cancer inpatients with COVID-19: A report from the Brazilian National Cancer Institute
Source: PLoS One. 2020 Oct 26;15(10):e0241261. doi: 10.1371/journal.pone.0241261 (PMC7588058; doi:10.1371/journal.pone.0241261)
Supplement: S2 Table — (DOCX) [file pone.0241261.s002.docx]

S2 Table. Specific therapies associated to the risk of death from Covid-19

| **Variables** | | **Alive** | **Death from COVID-19** | **OR (95%CI)** | **p-value** |  |
| --- | --- | --- | --- | --- | --- | --- |
| **Corticosteroids** | |  |  |  |  |  |
| No | | 50 (44·6) | 24 (40·0) | Ref· |  |  |
| Yes | | 62 (55·4) | 36 (60·0) | 1·2 (0·6-2·3) | 0·558 |  |
| Missing | |  |  |  |  |  |
| **Antibiotics** | |  |  |  |  |  |
| No | | 26 (23·2) | 5 (8·3) | Ref· |  |  |
| Yes | | 86 (76·8) | 55 (91·7) | 3·3 (1·2-9·2) | **0·020** |  |
| **Antiviral (Oseltamivir)** | |  |  |  |  |  |
| No | | 88 (78·6) | 44 (73·3) | Ref· |  |  |
| Yes | | 24 (21·4) | 16 (26·7) | 1·3 (0·6-2·8) | 0·439 |  |
| **Full anticoagulation** | |  |  |  |  |  |
| No | | 89 (79·5) | 45 (75·0) | Ref· |  |  |
| Yes | | 23 (20·5) | 15 (25·0) | 1·3 (0·6-2·7) | 0·502 |  |
| **Chloroquine** | |  |  |  |  |  |
| No | | 107 (96·4) | 56 (93·3) | Ref· |  |  |
| Yes | | 4 (3·6) | 4 (6·7) | 1·9 (0·5-7·9) | 0·373 |  |
| **Ivermectin** | |  |  |  |  |  |
| No | | 86 (76·8) | | 51 (85·0) | Ref· |  |
| Yes | | 26 (23·2) | | 9 (15·0) | 0·6 (0·3-1·3) | 0·205 |

Values in bold are statistically significant at p<0·05

*:172 patients included, 9 patients who died for other cancer related reasons were excluded from this mortality analysis.
